# Causes and characteristics of uveitis cases at a reference university hospital in Rio de Janeiro, Brazil

**DOI:** 10.5935/0004-2749.20220040

**Published:** 2022

**Authors:** Henrique Maciel Vieira de Moraes, Matheus Santos de Almeida, Karolyna Andrade de Carvalho, Ana Luiza Biancardi, Haroldo Vieira de Moraes Junior

**Affiliations:** 1 Serviço de Oftalmologia, Hospital Universitário Clementino Fraga Filho, Universidade Federal do Rio de Janeiro, Rio de Janeiro, RJ, Brazil

**Keywords:** Uveitis/epidemiology, Uveitis/etiology, Uveitis/ diagnosis, Toxoplasmosis, ocular, Hospital, university, Brazil/ epidemiology, Uveíte/epidemiologia, Uveíte/etiologia, Uveíte/ diagnóstico, Toxoplasmose ocular, Hospital universitário, Brasil/epidemiologia

## Abstract

**Purpose:**

The aim of this study was to describe the epidemiological profile of uveitis cases treated at University Hospital Clementino Fraga Filho and to identify the presentation pattern of intraocular inflammation on the basis of clinical, anatomical, etiological, and demographic criteria.

**Methods:**

A retrospective study was conducted using data from the medical records of 408 patients with active disease who attended the ophthalmology service between March and October 2018. Age, sex, visual acuity at the time of diagnosis, anatomical and etiological diagnoses, the clinical aspect, and the main symptoms reported during anamnesis were described.

**Results:**

Of the 408 patients in the study, 52% were male and 48% were female. The patients’ mean age was 42 years, and most (84%) were between 19 and 64 years old. Anterior uveitis was observed in 37.75% of the patients; posterior uveitis, in 49.75%; panuveitis, in 4.66%; and intermediate uveitis, in 3.43%. Only 18 patients (4.41%) presented with scleritis. Of the 390 patients with anatomical classifications, 76% had known etiologies, with the most prevalent diagnoses being toxoplasmosis (35.4%), followed by juvenile idiopathic arthritis (6.4%), ankylosing spondylitis (5.9%), and syphilis (4.9%). Infectious uveitis corresponded to 49.7% of the patients, while 26.6% of the cases were of noninfectious origin. Anterior uveitis had the highest number of cases classified as idiopathic (49.4%). In the cases of posterior uveitis, the etiology was established 94% of the time. The most frequent symptoms were ocular pain (71.8%) and blurring vision (56.8%).

**Conclusions:**

The present study confirmed the historical importance of infectious uveitis in our population, especially ocular toxoplasmosis. Uveitis appears to have no predilection for sex but mainly affects young people of working age, thus generating social and economic consequences. Despite the evolution of diagnostic methods, idiopathic uveitis remains one of the major etiologies. Epidemiological studies point to different presentation patterns of uveitis in different populations, but these may reflect the distinct characteristics of each institution.

## Abstract (pt)

**Objetivo:**

Descrever o perfil epidemiológico das uveítes atendidas no Hospital Universitário Clementino Fraga Filho - UFRJ. Identificando o padrão de apresentação da inflamação intraocular a partir de critérios clínicos, anatômicos, etiológicos e demográficos.

**Métodos:**

Estudo retrospectivo, com base em prontuários de 408 pacientes com doença ativa, atendidos no serviço de oftalmologia no período de março a outubro de 2018. Foram descritos a idade, sexo, acuidade visual no momento do diagnóstico, diagnóstico anatômico e etiológico, aspecto clínico, além dos principais sintomas relatados durante a anamnese.

**Resultados:**

Dos 408 pacientes do estudo, 52% eram do sexo masculino e 48% do feminino. A idade média dos pacientes foi de 42 anos, a maioria (84%) entre 19 e 64 anos. Uveíte anterior foi observada em 37,75% dos pacientes, uveíte posterior em 49,75%, panuveíte em 4,66% e uveíte intermediária em 3,43%; apenas 18 pacientes (4,41%) apresentaram diagnóstico de esclerite. Dos 390 pacientes com classificação anatômica, a etiologia foi determinada em 76% deles, com os diagnósticos mais prevalentes sendo Toxoplasmose (35,4%), artrite idiopática juvenil (6,4%), espondilite anquilosante (5,9%) e sífilis (4,9%). ) A uveíte infecciosa correspondeu a 49,7% desses pacientes, enquanto 26,6% eram de origem não infecciosa. A uveíte anterior teve o maior número de casos classificados como idiopáticos (49,4%), enquanto a uveíte posterior teve a etiologia estabelecida em 94% das vezes. Os sintomas mais frequentes foram dor ocular (71,8%) e visão embaçada (56,8%).

**Conclusões:**

O presente estudo confirmou a importância histórica da uveíte infecciosa em nossa população, principalmente a toxoplasmose ocular. As uveítes parecem não ter predileção por sexo, mas afetam principalmente jovens em idade ativa, gerando consequências sociais e econômicas. Apesar da evolução nos métodos diagnósticos, a uveíte idiopática continua sendo uma das principais causas. Estudos epidemiológicos apontam para diferentes padrões de uveíte nas populações, estes podem refletir características particulares de cada instituição.

## INTRODUCTION

Uveitis is the third leading cause and one of the leading causes of irreversible blindness in developed countries and worldwide, respectively^(1)^. Nevertheless, in many cases, it presents as a diagnostic and therapeutic challenge for general ophthalmologists and even specialists^(2)^.

In contrast to other eye conditions with potential morbidity, uveitis often develops in the age group of 20 to 50 years, and patients eventually abandon their studies and careers, thus generating a high socioeconomic impact and significant decline in quality of life^(3)^.

The heterogeneous distribution of uveitis cases worldwide is recognized when comparing studies performed with different populations and their respective risk exposures. Epidemiological studies that analyze the different types of uveitis and their respective etiologies play a fundamental role in providing subsidies that facilitate daily clinical practice^(4)^. Environmental, social, geographic, and genetic factors may be responsible for the distribution and main causes of uveitis in different populations and periods^(5)^. Despite the substantial impact of uveitis on public health, knowledge regarding the epidemiology of uveitis in developing countries, especially in Brazil, is limited^(6)^.

Most uveitis studies are conducted in developed countries, specifically in tertiary referral centers, resulting in proportions of uveitis distinct from those in most parts of the world and daily clinical practice^(7)^.

The purpose of the present study was to analyze and describe the epidemiological profile of uveitis cases trea ted at the Clementino Fraga Filho University Hospital of the Federal University of Rio de Janeiro (HUCFF-UFRJ). In addition, we identified the pattern of intraocular inflammation on the basis of clinical, anatomical, etiological, and demographic criteria.

## METHODS

A retrospective study, approved by the institutional ethical review board (CAAE: 93390618.6.0000.5257), based on an analysis of 408 medical records from new patients who attended the department of ophthalmology of the HUCFF-UFRJ, a public eye care reference service for referred patients from any levels of care all over the state, was conducted between March and October 2018.

All the patients had undergone an ophthalmological examination that consisted of detailed anamnesis, ocular ectoscopy, measurement of best-corrected visual acuity, applanation tonometry, anterior and posterior biomicroscopies, indirect ophthalmoscopy, and complementary evaluation methods when necessary, such as retinal imaging (ultrasonography, fluorescein angiography, and optical coherence tomography) and laboratory diagnostic tests.

The data of patients with uveitis who showed signs and symptoms of disease activity during follow-up were included in the statistical analysis. Uveitis cases presenting periceratic or limbic conjunctival hyperemia associated with the presence of cells and “flare” in the anterior chamber, ceramic precipitates, hypope, synechia, iris nodules, cells in the vitreous, snowballs, snowbanks, retinal or choroidal lesions, and vasculitis were considered active when associated with symptoms of pain, hyperemia, photophobia, decreased visual acuity, and floaters.

The following demographic and ophthalmological data where analyzed: age, sex, visual acuity at the time of diagnosis, anatomical and clinical classifications according to the established standard classification system^(8)^, etiological diagnosis, and the main symptoms reported by the patients.

The diagnostic criteria used for the main disease entities are briefly described as follows: toxoplasmosis was diagnosed when typical retinochoroiditis with active lesion, sometimes associated with old pigmented scars, and an enzyme-linked immunosorbent assay serological test result confirming the diagnosis; tuberculosis, when the Mantoux test result >15 mm, an epidemiological history of tuberculosis was present, and other entities were excluded; and herpes, when the clinical presentation was corroborated through serological tests. In more complex cases, polymerases chain reaction evaluation of the aqueous humor, collected through paracentesis, confirmed the hypothesis. The diagnoses of herpetic retinitis, acute retinal necrosis, and syphilis were based on the clinical presentation and corroborated through serological tests (human immunodeficiency virus [HIV], cytomegalovirus IgG and IgM, herpes IgG and IgG, hepatitis C, PPD, and syphilis). All the patients with systemic signs who attended the uveitis clinic of the ophthalmology department were evaluated by other clinical services at the same institution, such as the rheumatology and neurology departments, when the diagnosis had to be confirmed. Cases in which the cause of ocular inflammation was not identified, and a well-established clinical association was not possible were described as idiopathic uveitis.

The results collected underwent descriptive statistical treatment through percentages and were discriminated through tables and graphs. The statistical program SPSS version 22.0 (IBM, Armonk, NY) was used for data analysis. A Student *t* test and analysis of variance were applied for continuous variables. Relationships between categorical variables were analyzed using the Fisher exact test.

## RESULTS

A total of 408 patient records were analyzed and included in the study. The mean age was 42 years (range, 2-103 years), with the following age distribution (Table 1): between 0 and 18 years, 35 patients (8.5%); between 19 and 40 years, 167 patients (41%); between 41 and 64 years, 175 patients (43%); and older than 65 years, 31 patients (7.5%). Among all the patients, 213 (52%) were male and 195 (48%) were female.

**Table 1:** Uveitis distribution according to age and sex

Age range (years)	Male		Female		Total	
	n	%	n	%	n	%
0-18	15	7,04	20	10,25	35	8,57
19-40	95	44,6	72	36,93	167	40,93
41-64	88	41,32	87	44,62	175	42,9
≥65	15	7,04	16	8,2	31	7,6
Total	213	100	195	100	408	100

At the time of presentation, patients were divided into three categories according to the vision of the affected eye in unilateral uveitis and that of the eye with the best-corrected visual acuity in bilateral uveitis. In 170 cases (41.80%), the best-corrected visual acuity was between 20/20 and 20/50. In 112 cases (27.4%), the result corresponds to values between 20/60 and 20/200. Finally, in 126 cases (30.8%), the best-corrected visual acuity at the time of presentation was 20/400 or worse.

The symptoms most frequently reported by patients according to their medical records were eye pain and blurred vision, accounting for 71.8% and 56.8% of all complaints. In addition, ocular hyperemia (40.9%), photophobia (27.4%), floaters (4.3%), and photopsy (2.9%) were also recurrent complaints.

Of the 408 patients who surveyed, only 18 (4.4%) were diagnosed as having scleritis. Of the 18 patients, 11 (61%) were female and 7 (39%) were male, and 8 (44.5%) had anterior scleritis, 7 (39%) had posterior scleritis, and 3 (16.5%) had sclerouveitis.

Regarding the anatomical classification of the remaining 390 patients, the posterior uveitis was the most prevalent (203 cases, 49.75%), followed by anterior uveitis (154 cases, 37.75%), panuveitis (19 cases, 4.66%), and scleritis (18 cases, 4.41%). Finally, intermediate uveitis represented the smallest number of cases (only 14 cases, 3.43%).

Of the patients, 194 (49.74%) had the so-called infectious uveitis, of whom 175 (90.20%) had posterior uveitis, and 104 (26.66%) had noninfectious uveitis, of whom 62 (59.60%) had anterior uveitis. The remaining 92 cases (23.60%) were classified as idiopathic because their etiologies could not be determined. The results are presented in table 2.

**Table 2:** Uveitis distribution according to anatomical diagnosis and etiological origin

Primary site of inflammation	Infectious n (%)	Noninfectious n (%)	Idiopathic n (%)	Total^*^ n (%)
Anterior	16 (8,25)	62 (59,62)	76 (82,61)	154 (39,49)
Intermediate	- -	9 (8,65)	5 (5,43)	14 (3,59)
Posterior	175 (90,2)	17 (16,35)	11 (11,96)	203 (52,05)
Panuveitis	3 (1,55)	16 (15,38)	- -	19 (4,87)
Total	194 (100)	104 (100)	92 (100)	390 (100)

* Excluding 18 patients diagnosed as having scleritis.

Regarding the clinical aspect (Table 3), 210 cases (53.85%) of granulomatous uveitis were identified on the basis of the presence of toxoplasmosis and idiopathic uveitis with eye manifestations in 129 (61.42%) and 31 (14.76%), respectively. In 122 cases (31.28%), a non-granulomatous aspect was observed, among which 52 cases (42.62%) of uveitis corresponded to ocular manifestations of rheumatological diseases. A total of 58 cases (14.87%) did not have enough data in medical records regarding the clinical aspect of uveitis and thus remained unclassified.

**Table 3:** Diagnosis of uveitis classified according to clinical aspect

Diagnosis	Granulomatous	Nongranulomatous	Unclassified	Total^*^
Toxoplasmosis	129	0	9	138
Juvenile idiopathic arthritis	0	23	2	25
Ankylosing spondylitis	2	21	0	23
Syphilis	8	6	5	19
VKHS	14	0	2	16
Behcet disease	0	8	1	9
Pars planitis	2	1	5	8
Ceratite herpética	2	2	2	6
Tuberculosis	5	1	0	6
Acute retinal necrosis	5	0	1	6
Other	8	17	3	28
Idiopathic	31	34	27	92
Herpetic retinitis	4	0	1	5
Inflamatory bowel disease	0	5	0	5
Rheumatoid arthritis	0	4	0	4
**Total**	210	122	58	390

VKHS= Vogt-Koyanagi-Harada syndrome.

* Excluding 18 patients diagnosed as having scleritis.

As shown in table 4, the etiological diagnosis of uveitis was determined in 298 (76.40%) of the 390 cases. The most frequently found etiology was toxoplasmosis with 138 cases (35.40%), followed by juvenile idiopathic arthritis with 25 cases (6.42%), ankylosing spondylitis with 23 cases (5.90%), syphilis with 19 cases (4.90%), Vogt-Koyanagi-Harada syndrome (VKHS) with 16 cases (4.10%), Behcet disease with 9 cases (2.30%), and pars planitis with 8 cases (2.05%). Sharing the same position with 6 cases each (1.54%) were herpetic keratouveitis, tuberculosis, and acute retinal necrosis, followed by herpetic retinitis and inflammatory bowel disease with 5 cases each (1.30%) and rheumatic arthritis with 4 cases (1.03%). Sarcoidosis, endophthalmitis, and Hansen’s disease were present in 3 cases each (0.76%). Toxocoriasis, neuroretinitis, Blau syndrome, Fuchs’ heterochromic iridocyclitis, and Takayasu’s arteritis were present in 2 cases each (0.51%). Meanwhile, arboviral uveitis, cat scratch disease, diffuse unilateral subacute neuroretinitis, sporotrichosis, Posner’s syndrome, Krill’s disease, bilateral diffuse uveal melanocytic proliferation, carcinoma-associated retinopathy, and white dot syndrome were present in only 1 case each (0.25%).

**Table 4:** Distribuition of uveites etiology according to anatomical diagnosis

Etiology	All uveitis 390 (100%)	Anterior 154 (39,49%)	Intermediate 14 (3,59%)	Posterior 203 (52,05%)	Panuveitis 19 (4,87%)
Infections					
Toxoplasmosis	138			138	
Syphilis	19	6		13	
Herpetic keratouveitis	6	6			
Herpetic retinitis	5			5	
Tuberculosis	6			6	
Acute retinal necrosis	6			6	
Hanseniasis	3	3			
Endophthalmitis	3				3
Toxocariasis	2			2	
Neuroretinitis	2			2	
Arbovirosis	1			1	
Cat scratch disease	1			1	
DUSN	1			1	
Esporotricosis	1	1			
Subtotal	194 (49,74%)	16 (10,39%)	-	175 (86,21%)	3 (15,79%)
**Noninfectious**					
Juvenil idiopathic arthritis	25	25			
Ankylosing spondylitis	23	23			
VKHS	16				16
Behcet disease	9		1	8	
Pars planitis	8		8		
Inflamatory bowel disease	5	5			
Rheumatoid arthritis	4	3			
Sarcoidosis	3			3	
Blau syndrome	2	2			
Fuchs heterochromic iridocyclitis	2	2			
Takayasu arteritis	2			2	
Posner Schlossman syndrome	1	1			
Retinal pigment epithelitis	1			1	
BDUMP	1			1	
CAR syndrome	1			1	
White-dot syndrome	1			1	
Subtotal	104 (26,67%)	62 (40,26%)	9 (64,29%)	17 (8,37%)	16 (84,21%)
Idiopathic	92 (23,59%)	76 (49,35%)	5 (35,71%)	11 (5,42%)	-

DUSN= diffuse unilateral subacute neuroretinitis; VKHS= Vogt-Koyanagi-Harada syndrome; BDUMP= bilateral diffuse uveal melanocytic proliferation; CAR= carcinoma associated retinophaty.

## DISCUSSION

This retrospective study evaluated the distribution of uveitis cases treated at the university hospital of the School of Medicine of the Federal University of Rio de Janeiro. As a retrospective study, through the analysis of medical records, a minimal loss of patients due to unavailability of data was inevitable. In addition, data were not available throughout the calendar year. Besides treating patients from all over the state, the ophthalmology service of the institution receives patients with signs of eye inflammation referenced directly from the city’s main ophthalmological emergency service, the *Hospital Municipal Souza Aguiar*.

Despite the diversity in the origin of patients included in our study in contrast to those in most hospital-based studies on uveitis that were conducted in specialized centers around the world, we recognized that cases from a tertiary center do not truly represent the population, reflecting an overview of only the most complicated and challenging cases in each region.

Nevertheless, we understand the importance of these surveys, especially in countries such as Brazil, as they contribute to the identification of the local epidemiological profile of uveitis, being capable of directing the need for public health policies by reinforcing the clinical scenario and notoriety of many etiologies.

For comparison, table 5 presents an analysis of the epidemiological characteristics of several studies on uveitis that have been conducted in recent decades in different regions worldwide, with their respective populations, including the results of this work.

**Table 5:** Comparative of uveitis epidemiology in different locations

Study^*^	Year	Location	Sex (M:F)	Age (average)	Patients (n)	AU (%)	IU (%)	PU (%)	Panuveitis (%)	Institution (level of care)
Mc Cannel^9^	1996	CA, USA	1:1	45	213	60,6	12,2	14,6	9,4	Tertiary
Oruc^12^	2003	KY, USA	1:1	46	853	22,3	10,9	48,4	18,4	Tertiary
Gouveia^10^	2004	SP, Brazil	1:1,5	41	262	20	4,5	39,7	31,3	Tertiary
DAS^17^	2009	Assam, India	2,1:1	32,5	308	47,1	13	29,9	10	Tertiary
Kawashima^13^	2010	Saitama, Japan	1:1,2	49	474	44,3	-	9,7	40,1	Tertiary
Silva^19^	2013	SP, Brazil	1:1	25,5	812	0,3	0,1	91,9	3,1	Tertiary
Camilo^11^	2014	PE, Brazil	1,7:1	43	117	70,1	0	26,5	3,4	Primary
Grajewski^14^	2015	Cologne, Germany	1,1:1	45	474	53	19	21	7	Tertiary
Fernandez^15^	2016	SP, Brazil	1:1,2	40	1053	29,2	7,22	40,1	15,9	Tertiary
Moraes^**^	2020	RJ, Brazil	1,1:1	42	408	37,7	3,4	49,8	4,6	Tertiary

M= male; F= female; AU= anterior uveitis; IU= intermediate uveitis; PU= posterior uveitis;

* first name of author;

** 4,5% of patients in the present study are classified as scleritis and are not represented in the table.

In parallel with most studies worldwide^(9-15)^, the current survey shows a greater involvement of individuals during their most productive life, between 19 and 64 years of age, with 84% of our patients generating real socioeconomic cost^(16)^. We also found no significant difference in sex distribution in contrast to that observed in predominantly agricultural communities with difficult access to medical care, as reported by Das et al. in their work on an Indian referral center^(17)^.

When considering the anatomical distribution of uveitis cases, geography seems to represent an important factor in the epidemiology of the disease, as exemplified in Table 5. In contrast to most international studies, especially in Western countries, anterior uveitis accounts for most cases, the present study along with previous others conducted in national territory shows posterior uveitis as the main form of presentation^(18)^. This disparity occurs in many developing nations owing to the high number of cases related to infectious etiologies such as toxoplasmosis in Brazil and tuberculosis in India^(17,19)^. Such entities can cause severe and permanent damage to the retina, resulting in significant vision impairment.

In our patients, infectious uveitis accounted for almost 50% of cases. When isolating this group, the most prevalent etiology, accounting for 71% of diagnoses, was toxoplasmosis. National surveys, regardless of the period, demonstrate the importance of this disease in our country. Whether by frequency in our population, the ability to affect any age group, or even severity, toxoplasmosis and its consequences pose a major threat to visual health, thus requiring prevention, early diagnosis, and appropriate treatment. The distribution of the main causes of uveitis in our analysis and other populations in important studies conducted in Brazil and abroad is presented in table 6.

**Table 6:** Characteristics from the main diagnosis of uveitis in different studies (%)

	Moraes RJ, Brasil 2020	Mc Cannel^9^ CA, USA 1996	Oruc^12^ KY, USA 2003	Gouveia^10^ SP, Brazil 2004	DAS^17^ Assam, India 2009	Kawashima^13^ Saitama, Japan 2010	Silva^19^ SP, Brazil 2013	Camilo^11^ PE, Brazil 2014	Grajewski^14^ Cologne, Germany	Fernandez^15^ SP, Brazil 2016
**Diagnosis**	**408**	**213**	**853**	**262**	**308**	**474**	**812**	**812**	**474**	**1053**
Toxoplasmosis	33,8	4	3,9	21,6	12	0,8	88,7	21,3	7,1	24
Juvenile idiopathic arthritis	6,1	1,3	0,4	4,5	11	-	-	-	1,9	6,3
Ankylosing spondylitis	5,6	-	1	3,8	-	-	-	-	-	-
Syphilis	4,6	0,4	-	1,1	0,3	-	0,2	-	-	6,1
VKHS	3,9	0,4	0,4	12,6	4,5	3,4	1,3	1,7	0,6	7,5
Behcet disease	2,2	0,8	0,2	9,9	0,3	4,6	1,4	-	1,9	3,5
Pars planitis	1,96	-	8,7	2,7	10	-	0,1	-	-	4,7
Tuberculosis	1,,5	-	-	3,8	2,9	0,4	-	-	-	5,2
Hanseniasis	0,7	-	-	0,4	-	-	0,3	-	-	0,4
Sarcoidosis	0,7	1	2,2	2,3	4,5	7,2	-	-	11,3	2,3
HLA-B27 associate	-	17,1	-	-	-	-	-	-	10,1	6,3
Idiopathic	22,5	52,6	30,7	19,4	28,8	47,5	3,9	61,5	40,9	9,9

VKHS= Vogt-Koyanagi-Harada syndrome; HLA= human leukocity antigen.

We also suggest the importance of other infectious causes such as tuberculosis, of which little is known about its extrapulmonary form in the general population; herpes; and syphilis, which represents the second most frequently diagnosed infectious etiology in our service that is especially dangerous because of its generic presentation capacity.

Only patients with posterior infectious uveitis or panuveitis were tested for HIV. Of these patients, only 2 tested positive, both having a CD4 count of >350.

Regarding noninfectious uveitis, the incidence rates of cases associated with rheumatological systemic diseases, especially juvenile idiopathic arthritis and ankylosing spondylitis, were significantly high, representing the second and third most prevalent diagnoses on our entire sample, respectively.

Other noninfectious causes included Behçet’s disease and VKHS. Despite the significant number of cases, surveys conducted in the population of São Paulo, especially the capital, showed an even greater importance of the two etiologies, as shown in Table 6. This difference may be related to the large Japanese-Brazilian community historically established in the most populous city in the country^(20)^.

Uveitis that was not etiologically diagnosed and then classified as idiopathic represent >20% of all our cases, regardless of anatomical classification. This finding is compatible with and, in some cases, inferior (as shown in Table 6) to those of other similar studies worldwide. In relation to anterior uveitis, idiopathic causes were mainly responsible for almost half of all cases. By contrast, posterior uveitis could be accurately diagnosed in approximately 94% of cases, probably owing to the presence of characteristic ophthalmologic findings present in these conditions, and best exemplified by toxoplasmosis in the present study.

Our study analyzed the distribution of uveitis cases in a referral service in the state of Rio de Janeiro. We found high prevalence rates of infectious and posterior uveitis as compared with those in other studies worldwide, probably associated with the historical presence of to xoplasmosis in our population and the diagnosis of rheumatological systemic diseases that generate ophthalmic manifestations in the form of intraocular inflammation such as juvenile idiopathic arthritis and ankylosing spondylitis.

In the present study uveitis did not present a predilection for sex; however, it mainly affected the young and adult working population, thus generating social and economic consequences.

Despite the evolution of diagnostic methods, no definite diagnosis was established in almost one-fifth of our sample, making idiopathic uveitis one of the main causes, especially in those whose primary site of inflammation was the anterior segment of the eye. Our results may help to identify the epidemiological profile of uveitis in Brazil, setting our priorities regarding prevention and public health.
